# Manual versus Pump Infusion of Distending Media for Hysteroscopic Procedures: A Randomized Controlled Trial

**DOI:** 10.1038/s41598-019-51252-3

**Published:** 2019-10-18

**Authors:** Wan-Hua Ting, Ho-Hsiung Lin, Sheng-Mou Hsiao

**Affiliations:** 1 Department of Obstetrics and Gynecology, Far Eastern Memorial Hospital, New Taipei, Taiwan; 2 Department of Obstetrics and Gynecology, National Taiwan University College of Medicine and National Taiwan University Hospital, Taipei, Taiwan; 3 Graduate School of Biotechnology and Bioengineering, Yuan Ze University, Taoyuan, Taiwan

**Keywords:** Therapeutic endoscopy, Randomized controlled trials

## Abstract

Fluid overload is a potential complication of hysteroscopic procedures with the possibility of dangerous electrolyte changes. This prospective randomized controlled trial aimed to compare perioperative outcomes and changes in electrolytes after hysteroscopic procedures between the manual infusion (MI) and the pump infusion (PI) methods for distending media infusion. One hundred consecutive women who had hysteroscopic procedures between December 2013 and February 2017 were recruited and randomly allocated to either the MI or PI group. The PI group was associated with an increased volume of infused fluid and collected fluid compared with the volumes of the MI group. Almost all serum electrolyte levels differed significantly between the baseline and postoperative values in both groups; however, no significant differences were noted between the groups. The change in potassium level was positively correlated with the volume of fluid deficit (Spearman’s rho = 0.24, P = 0.03), whereas the change in calcium level was negatively correlated with the volume of fluid deficit (Spearman’s rho = −0.26, P = 0.046). With no between-group differences in the changes in the other perioperative parameters and electrolytes, the MI method can be a good alternative for delivering distending media for hysteroscopic procedures.

## Introduction

Fluid overload is a life-threatening complication in hysteroscopic procedures. Excessive absorption of distending media, especially hypotonic, electrolyte-free media, may result in dilutional hyponatraemia, potentially causing cerebral oedema, coma and death^1^. Any type of distending media can potentially cause the above complications following rapid systemic absorption.

Technological advancements in recent years have resulted in the development of bipolar electrodes^2^. Studies comparing unipolar and bipolar electrodes support the safety profile of bipolar electrodes in hysteroscopic surgeries^3–5^. However, in locations where bipolar electrodes are unavailable, various methods have been adopted to reduce the incidence of fluid overload, including automated fluid monitoring, preoperative use of vasopressin or gonadotropin-releasing hormone agonists^1^.

In a previous retrospective study, manual infusion (MI) of distending media was associated with a significantly reduced average volume of infused fluid, reduced operative time and lower postoperative abdominal pain scores compared with the pump infusion (PI) method^6^. However, the study results might be biased given its retrospective nature^6^. Thus, the primary objective of this prospective randomized controlled study is to compare the infused volume of the MI method with the PI method in hysteroscopic procedures. The secondary objective of this study was to compare the other perioperative outcomes and postoperative changes in serum electrolytes and blood osmolarity between the MI and PI groups.

## Results

**Figure 1:**
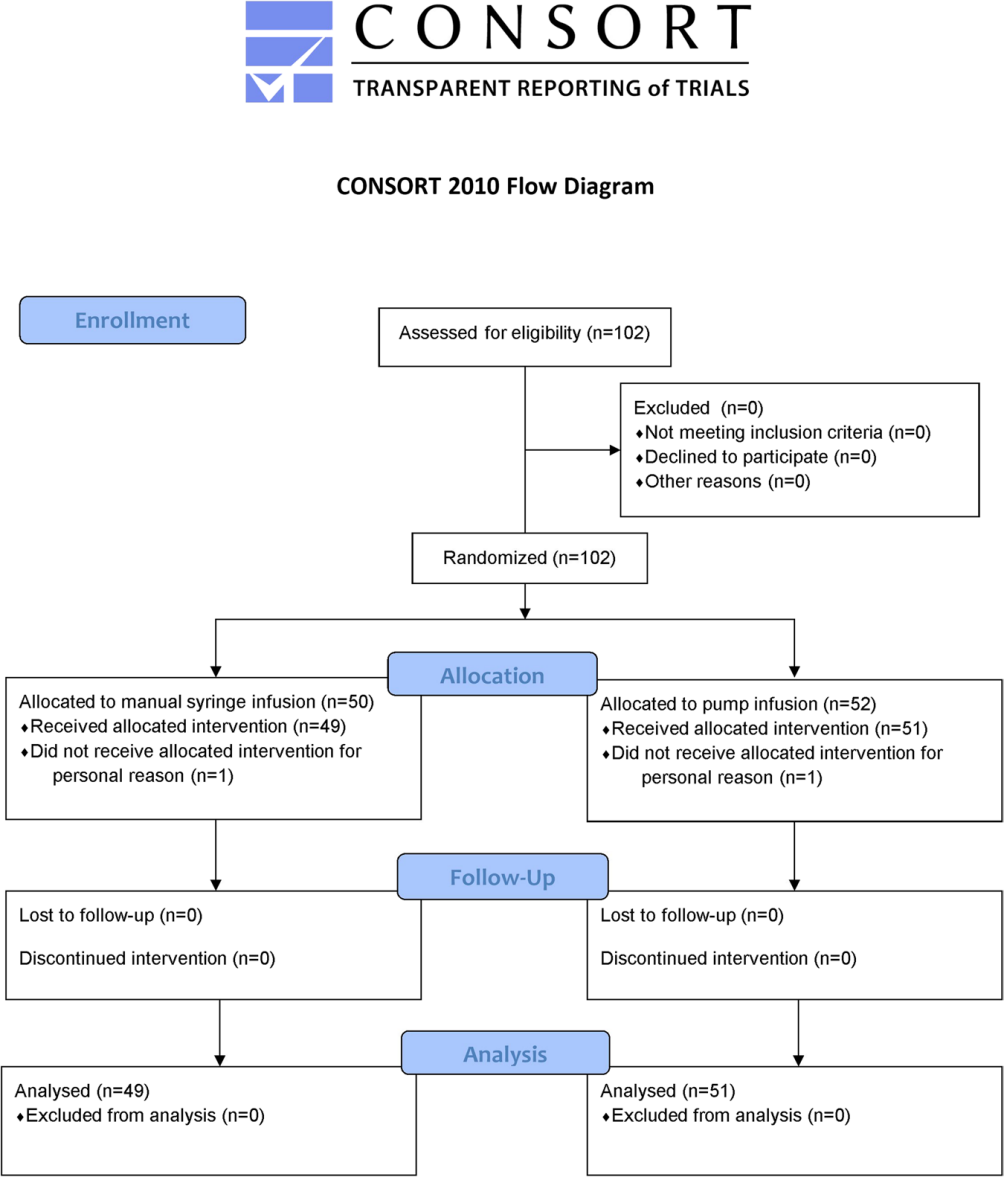
Flowchart of the participants who underwent hysteroscopic procedures^31^.

One hundred and two consecutive women were enrolled in this study. Two women did not receive allocated interventions for personal reasons. Forty-nine women in the MI group and 51 women in the PI group underwent hysteroscopic procedures (Fig. 1). There were 12 women receiving hysteroscopic myomectomy in the MI group and 11 women receiving hysteroscopic myomectomy in the PI group (Table 1).

**Table 1:** Comparison of the baseline characteristics and perioperative data of women in the manual infusion group and the pump infusion group (n = 100) and myomectomy cases (n = 23).

Variables	All cases			Myomectomy cases		
	Manual infusion group (n = 49)	Pump infusion group (n = 51)	^†^P	Manual infusion group (n = 12)	Pump infusion group (n = 11)	^†^P
Age (years)	44.6 ± 8.8	46.5 ± 8.1	0.29	46.7 ± 6.3	49.9 ± 6.3	0.23
Body mass index (kg/m2)	23.5 ± 4.5	22.8 ± 3.2	0.62	26.5 ± 6.5	22.0 ± 2.7	0.06
Parity	1.4 ± 1.0	1.9 ± 0.9	0.01	1.6 ± 1.1	1.8 ± 0.7	0.65
Diagnostic hysteroscopy	10 (20)	8 (16)	0.48	—	—	
Operative hysteroscopy	39 (80)	43 (84)	0.48	—	—	
Haemoglobin (g/dL)	11.6 ± 2.3	10.7 ± 2.4	0.14	11.1 ± 2.9	10.6 ± 1.9	0.64
Serum K level (mmol/L)	4.1 ± 0.3	4.1 ± 0.4	0.48	4.0 ± 0.3	4.0 ± 0.5	0.73
Serum Na level (mmol/L)	140.4 ± 2.2	140.1 ± 2.4	0.34	140.3 ± 2.2	139.1 ± 3.2	0.46
Serum Cl level (mmol/L)	103.6 ± 2.4	103.5 ± 3.1	0.68	104.5 ± 1.7	104.5 ± 5.0	0.63
Serum Ca level (mg/dL)	9.1 ± 0.4	9.2 ± 0.4	0.44	9.0 ± 0.3	9.1 ± 0.4	0.86
Serum P level (mg/dL)	3.3 ± 0.5	3.4 ± 0.5	0.40	3.0 ± 0.4	3.0 ± 0.7	1.00
Serum Mg level (mmol/L)	2.0 ± 0.2	2.1 ± 0.2	0.12	2.0 ± 0.2	2.2 ± 0.2	0.17
Blood osmolality (mmol/kg)	288.7 ± 4.5	288.5 ± 4.6	0.87	286.0 ± 3.2	289.8 ± 6.4	0.33
The largest diameter of myoma (cm)	—	—	—	2.8 ± 1.1	3.3 ± 1.4	0.52
Infused fluid (mL)	981 ± 639	1355 ± 789	0.009	1419 ± 976	1647 ± 715	0.34
Collected fluid (mL)	793 ± 602	1221 ± 714	0.001	1233 ± 956	1568 ± 707	0.14
Fluid deficit (mL)	187 ± 180	134 ± 227	0.22	187 ± 158	80 ± 335	0.64
Estimated blood loss (mL)	26.5 ± 34.1	27.8 ± 50.7	0.35	45.4 ± 54.3	69.1 ± 96.0	0.80
Operative time (min)	19.4 ± 10.1	17.7 ± 7.4	0.78	28.0 ± 14.8	21.8 ± 7.8	0.46
First postoperative pain score (0–10)	0.69 ± 1.12	0.51 ± 0.83	0.48	0.55 ± 0.93	0.45 ± 0.93	0.69
Second postoperative pain score (0–10)	0.71 ± 0.73	0.34 ± 0.55	0.008	0.82 ± 0.75	0.36 ± 0.50	0.13

Values are expressed as the mean ± standard deviation or number (percentage).

^†^Wilcoxon rank-sum test or chi-square test.

**Figure 2:**
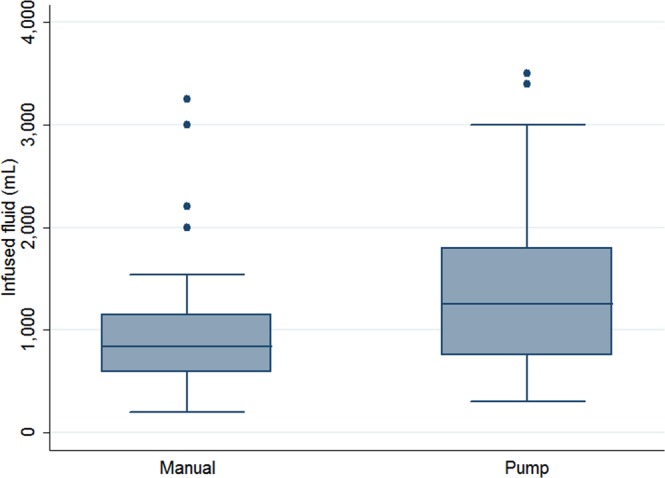
Box plot of the volume of infused fluid between the pump infusion and manual infusion groups.

Except for parity, there were no significant differences in the baseline characteristics between the two groups of women (Table 1). The volumes of infused fluid and collected fluid of the MI group were significantly reduced compared with those of the PI group (infused fluid: 981 ± 639 vs. 1355 ± 789, P = 0.009, Fig. 2; collected fluid: 793 ± 602 vs. 1221 ± 714, P = 0.001, Table 1). In addition, a higher second postoperative pain score was found in the MI group compared with the PI group (0.71 ± 0.73 vs. 0.34 ± 0.66, P = 0.008, Table 1).

All women completed the surgical procedure as scheduled, and there were no failed cases. None of the women in either group experienced perioperative complications related to surgery. Eight women (5 from the PI group, 3 from the MI group) had blood transfusion due to preoperative symptomatic anaemia. In the MI group, one woman with menorrhagia was found to have a grade 3 endometrioid endometrial cancer after hysteroscopic myomectomy. She subsequently underwent a robotic staging operation, and her pathological staging was International Federation of Gynecology and Obstetrics stage 1 A with negative peritoneal washing cytology. She remained alive without evidence of disease.

Almost all serum electrolyte levels differed significantly between the baseline and postoperative values in both groups; however, no significant differences were noted between the groups (Table 2). In the subgroup analysis of myomectomy cases, the changes in potassium levels and magnesium levels from baseline were significantly greater in the PI group than those in the MI group (Table 2). None of the women had postoperative serum sodium levels less than 130 mmol/L. The change in potassium level was positively correlated with the volume of the fluid deficit (Spearman rho = 0.24, P = 0.03, Table 3), whereas the change in calcium level was negatively correlated with the volume of fluid deficit (Spearman rho = −0.26, P = 0.046, Table 3).

**Table 2:** Comparisons of the changes in serum electrolyte levels, blood osmolarity, haemoglobin and pain between the pump infusion and the manual infusion groups (n = 100) and myomectomy cases (n = 23).

Variables	All cases				Myomectomy cases			
	^†^Manual infusion group (n = 49)	^†^Pump infusion group (n = 51)	^‡^P	^§^Power	^†^Manual infusion group (n = 12)	^†^Pump infusion group (n = 11)	^‡^P	^§^Power
Change in serum K level (mmol/L)	−0.1 ± 0.5	−0.2 ± 0.4**	0.48	0.11	0.1 ± 0.5	−0.4 ± 0.4*	0.04	0.65
Change in serum Na level (mmol/L)	−1.1 ± 2.6**	−0.5 ± 3.0	0.21	0.17	−0.6 ± 2.6	1.1 ± 1.5	0.10	0.47
Change in serum Cl level (mmol/L)	1.4 ± 2.9**	1.8 ± 3.5**	0.66	0.08	3.1 ± 2.9*	3.3 ± 5.4	0.88	0.05
Change in serum Ca level (mg/dL)	−0.7 ± 0.5**	−0.8 ± 0.7**	0.54	0.10	−0.7 ± 0.6*	−1.3 ± 1.3*	0.47	0.18
Change in serum P level (mg/dL)	0.5 ± 0.6**	0.2 ± 0.5	0.21	0.33	0.5 ± 0.6	0.4 ± 0.7	1.00	0.06
Change in serum Mg level (mmol/L)	−0.1 ± 0.2**	−0.2 ± 0.2**	0.11	0.46	0.0 ± 0.4	−0.4 ± 0.3*	0.03	0.62
Change in blood osmolality (mmol/kg)	−0.8 ± 12.7*	−2.5 ± 3.6**	0.64	0.11	6.3 ± 24.2	0.0 ± 1.4	0.16	0.11
Change in haemoglobin (g/dL)	−0.7 ± 1.0**	−0.7 ± 1.3**	0.76	0.05	−0.4 ± 1.0	−1.2 ± 1.8	0.32	0.21

^†^Within-group comparisons were performed by the Wilcoxon signed-rank test, and *P < 0.05, **P < 0.01.

^‡^Wilcoxon rank-sum test.

^§^Significance level of the test = 0.05.

**Table 3:** Correlations between the fluid deficit and postoperative changes in serum electrolyte levels (n = 100).

Variables	^†^ρ for fluid deficit	^†^P
Change in K (mmol/L)	0.24	0.03
Change in Na (mmol/L)	−0.12	0.27
Change in Cl (mmol/L)	−0.17	0.18
Change in Ca (mg/dL)	−0.26	0.046
Change in P (mg/dL)	−0.15	0.34
Change in Mg (mmol/L)	−0.04	0.79
Change in osmolality (mmol/kg)	−0.02	0.86

Values are expressed as the mean ± standard deviation.

^†^Each variable was compared with the fluid deficit by the Spearman rank-correlation test.

## Discussion

In this study, the MI group had a significantly lower volume of infused fluid and collected fluid compared with the PI group (Fig. 2), which was consistent with the results of a previous study^6^. The continuous flow based on pressure by an automatic pump in the PI group instead of the intermittent flow based on visibility in the MI group might explain the above finding of higher infused and collected fluid volumes in the PI group. Theoretically, the greater the infused volume, the greater the risk of excessive fluid absorption will be, as concluded in the findings of the previous study^6^. Wong *et al*., who evaluated the efficacy of transcervical injection of vasopressin during hysteroscopic myomectomy, found a positive correlation between the infused volume and fluid deficit^7^. We also found a significant correlation between the infused volume and fluid deficit (Spearman’s rho = 0.27, P = 0.008) in the current study; therefore, it is advised to keep the amount of infused volume as low as possible.

The change in potassium level was positively correlated with the volume of fluid deficit in this study, which could be explained as a consequence of haemolysis or intracellular uptake of the infused fluid^8,9^. Haemolysis can occur either with the use of distilled water, which is extremely hypotonic, or iatrogenically during the collection of blood samples^8,10^. Hyperkalaemia, especially a serum potassium level of >6 mEq/L, may result in life-threatening arrhythmia. Gupta *et al*. reported that the percentage increase in serum potassium may be up to 16.19% if a large amount of infused fluid was used during the transurethral resection of the prostate (TURP) procedure^9^. Panovska Petrusheva *et al*. also reported an increase in the potassium level after the TURP procedure that was associated with the amount of infused fluid^11^. The TURP procedure is similar to the operative hysteroscopic procedure. Thus, the serum potassium level should also be reassessed if a large fluid deficit was noted during the hysteroscopic procedure.

Our study revealed a negative correlation between the change in calcium level and the fluid deficit volume (Table 3). The dilution of plasma induced by excessive systemic absorption is a possible explanation^12^. Severe hypocalcaemia may be associated with seizures, hypotension, heart failure, or laryngospasm. Lee *et al*. reported that hypotension occurred in a woman with TURP syndrome (i.e., hyponatraemia and associated symptoms following TURP or hysteroscopy) due to a reduced ionized calcium concentration during hysteroscopy, and her refractory hypotension improved after 40 mL of 3% calcium chloride^12^. Agarwal *et al*. also reported a case of TURP syndrome with hypocalcaemia after the TURP procedure^13^. Thus, our findings support the coexistence of hypocalcaemia in women with large fluid deficits after hysteroscopic or TURP procedures^12,13^. Although hyponatraemia rarely develops in women who received a bipolar hysteroscopic procedure, it is prudent to assess the serum calcium level if a large fluid deficit occurred.

In our study, although the serum sodium concentrations were significantly decreased in the MI group after surgery (Table 2), the average reduction in serum sodium was only 1.1 ± 2.6 mmol/L. Tammam *et al*. reported a decrease of 4.77 ± 0.831 mmol/L in serum sodium levels after unipolar hysteroscopic surgeries^4^. Similarly, Berg *et al*. reported a reduction in the mean serum sodium to 133.8 mmol/L from 138.8 mmol/L in the unipolar group^5^. Nonetheless, the operative time was markedly longer in Tammam *et al*.’s study (31.93 ± 12.92 minutes) compared to that in our study (19.4 ± 10.1 minutes, Table 1), and the infused fluid volume in Berg *et al*.’s study was notably higher (3463 ± 1435 mL) compared to that in our study (981 ± 639 mL, Table 1)^4,5^. These differences could partly explain the lower changes in serum sodium levels in our MI group.

In our study, a higher second postoperative pain score was found in the MI group compared with that in the PI group (0.71 ± 0.73 vs. 0.34 ± 0.66, P = 0.008, Table 1). The above finding might be related to the transient higher in-flow pressure applied by the assistant during the MI procedure compared with that of the PI method, which has a constant in-flow pressure of 70–100 mmHg^6,14,15^.

In order to reduce the time required for hysteroscopy, we performed hysteroscopic myomectomy by resection of the myoma pedicle, followed by fractioning the myoma into smaller portions and subsequent myoma extraction by a pair of forceps, as described by Lin *et al*.^16,17^. Approximately one-third of the myoma was removed using the resectoscope, and two-thirds was removed using forceps with the help of sonographic guidance in their study^17^. In our study, although the MI method provided only intermittent visualization for the hysteroscopic procedure, it was adequate for hysteroscopic resection of the myoma pedicle and fractioning of the myoma.

Hysteroscopic myomectomy is a complicated surgical procedure that may lead to serious complications and requires a long learning curve. Some innovations have been introduced to overcome the limitations of conventional hysteroscopic myomectomy, including the introduction of hysteroscopic tissue removal systems (i.e., hysteroscopic morcellators), such as the Truclear device (Medtronic, Minneapolis, Minnesota, USA) and the MyoSure device (Hologic, Marlborough, Massachusetts, USA)^18,19^. Both devices were shown to be effective in hysteroscopic myomectomy^18–21^. However, life-threatening complications such as fluid overload, uterine perforation and bleeding do occur with hysteroscopic morcellations^22^.

In addition, preoperative use of dienogest, an orally administrable progestin, has good endometrial thinning effects and tolerable side effects^23,24^. Nevertheless, the choice for hormonal endometrial preparation before hysteroscopic surgery requires robust cost-effectiveness analyses^23^.

In our study, due to the unavailability of 3% sorbitol and 1.5% glycine in our institution, distilled water was used for unipolar hysteroscopic myomectomy^6,25^. Distilled water was the original distending medium for resectoscopic urologic surgery^14^. Nonetheless, it is worth mentioning that if distilled water is absorbed systemically, one of the risks was haemolysis. The addition of solutes such as sorbitol and glycine increased the medium’s osmolality to a degree that haemolysis could be largely prevented^14^.

Electrolyte-free low viscosity solutions, such as 3% sorbitol and 1.5% glycine, have been widely used as distending media during hysteroscopic surgeries using unipolar instruments^26–28^. Nonetheless, intraoperative absorption of these electrolyte-free fluids, including distilled water, 3% sorbitol and 1.5% glycine, can cause hyponatraemia, hypoosmolality, volume overload and pulmonary oedema^26,27^. It is important to meticulously monitor fluid balance and reduce operation time to minimize complications.

Gravity is the simplest method to deliver the distending medium into the intrauterine cavity^14^. The in-flow pressure is approximately 70 to 100 mmHg when the bag with distending medium is 1 to 1.5 m above the uterus^14,15^, and the height should be kept at the minimum elevation to allow sufficient uterine distention. It has been demonstrated that systemic absorption is greater with increasing intrauterine pressure, especially if it exceeds mean arterial pressure^14^. The mean arterial pressure in normal healthy people without known cardiovascular risk factors has been reported to be 88 ± 9 mmHg^29^. Hasham *et al*. also reported that there was no absorption of the distending medium into the venous system at an intrauterine pressure of 70 mmHg, while at 150 mmHg, contrast distending medium was clearly seen entering the uterine venous plexus^30^. Thus, the in-flow pressure of 70–100 mmHg in our PI group seems to be reasonable.

The main limitation of this study is the limited number of hysteroscopic myomectomy procedures. However, the randomized study design might compensate for this limitation. In addition, although there were statistically significant between-group differences in the infused and collected fluid volumes, owing to their limited powers (Table 2), the interpretation of the between-group differences in the fluid deficit, serum electrolytes and blood osmolality should be made with caution.

In conclusion, the MI method was associated with a reduced infused fluid compared with that of the PI method. With no between-group significant differences in the changes in the other perioperative parameters and electrolytes, the MI method can be a good alternative for delivering distending media for less complex hysteroscopic procedures.

## Materials and Methods

This study was performed at the Department of Obstetrics and Gynecology of Far Eastern Memorial Hospital and was approved by the Research Ethics Committee of the institution. All women aged 20 years and older who had indications for hysteroscopic procedures between December 2013 and February 2017 were invited to participate in the study. Before receiving the intervention, all women provided written informed consent. All methods were performed in accordance with the guidelines and regulations specified by Far Eastern Memorial Hospital.

### Surgical procedure

Women were allocated to the MI or PI group in a randomized order based on computer-generated random numbers at a ratio of 1:1. The surgical procedures were performed by the corresponding author. Under intravenous anaesthesia, cervical dilation was performed with Hegar uterine dilators, followed by insertion of a unipolar resectoscope with an outer diameter of 8 mm (Karl Storz, Tuttlingen, Germany) into the uterine cavity. The uterine cavity was then infused with distilled water for inspection, resection, or ablation of tissue. In the MI method, a 60-mL disposable syringe (BD Plastipak TM, BD Medical, County Louth, Ireland) was connected to the resectoscope via a 90-cm extension tube (Sigma, Sigma Medical Supplies Corp., Taiwan), and an assistant helped to pump the distilled water manually. Two 60-mL syringes were used at one time to minimize the waiting time required for refilling the syringe. A large collecting bag was tucked beneath the woman’s gluteal region and secured to the surgeon’s gown to capture fluid spilled from the cervix and the resectoscope. The total volumes of infused fluid and outflow fluid were recorded. In the PI method, a continuous-flow fluid infusion pump device (1-L Pressure Infuser Irrigation Pump, ConMed, Utica, New York, USA) was used to deliver the fluid media at a constant in-flow pressure of 70–100 mmHg, depending on the visibility of the surgical view, and the remaining procedures were performed in a manner similar to that of the MI group^6^.

### Measurements

Blood samples were drawn before and after surgery. The blood sample was sent for analysis of complete blood count, blood osmolarity and serum electrolytes, including sodium, potassium, chloride, magnesium, calcium and phosphorus. The women’s characteristics, clinical indications for the hysteroscopic procedure, the volume of distending media used, and perioperative data were recorded. The fluid deficit was calculated by subtracting the total volume of collected fluid media from the total infused volume. The postoperative pain scores (0–10) were recorded twice by self-reporting using a visual analogue scale at baseline when the patient recovered from anaesthesia in the recovery room (i.e., first postoperative pain score) and 30 minutes later prior to discharge from the recovery room (i.e., second postoperative pain score). Changes in serum electrolytes, blood osmolarity and haemoglobin were determined by subtracting the preoperative measurements from the postoperative measurements.

### Statistical analysis

The Wilcoxon rank-sum test and the Chi-square test were employed for statistical analyses using STATA software (Version 11.0; Stata Corp, College Station, TX, USA). A p-value of less than 0.05 was considered statistically significant.

Given that the primary objective of this study is to compare the infused volume between the MI and PI groups, the variable was used to calculate the sample size required. A previous study reported an infused volume of 1117 ± 712 mL in the MI group and 2216 ± 1502 mL in the PI group^6^. To detect a difference in the amount of infused fluid, we conducted a test with a significance level of 0.05 and a power of 0.9. We anticipated that two groups of equal size would be required. Thus, we concluded that at least 25 subjects in each group were required to test the above hypothesis.

### Trial registration

NCT02012010, registered on 16/12/2013. Available at ClinicalTrials.gov, www.clinicaltrials.gov.

Clinical Trial Registration Number and date of registration: ClinicalTrials.gov, www.clinicaltrials.gov, NCT02012010, 16/12/2013. Number of IRB and date of approval: 102124-F, 21/10/2013.

## Supplementary information

CONSORT 2010 checklist

Research Protocol

## Data Availability

The datasets generated and/or analysed during the current study are available from the corresponding author on reasonable request.

## Supplementary information

is avilable for this paper at 10.1038/s41598-019-51252-3.

## References

[1] Munro MG, Christianson LA (2010). Complications of hysteroscopic and uterine resectoscopic surgery. Obstel. Gynecol. Clin. North. Am..

[2] Mencaglia L, Lugo E, Consigli S, Barbosa C (2009). Bipolar resectoscope: The future perspective of hysteroscopic surgery. Gynecol. Surg..

[3] Roy KK (2015). Hysteroscopic septal resection using unipolar resectoscope versus bipolar resectoscope: Prospective, randomized study. J. Obstet. Gynaecol. Res..

[4] Tammam AE, Ahmed HH, Abdella AH, Taha SAM (2015). Comparative study between monopolar electrodes and bipolar electrodes in hysteroscopic surgery. J. Clin. Diagn. Res..

[5] Berg A, Sandvik L, Langebrekke A, Istre O (2009). A randomized trial comparing monopolar electrodes using glycine 1.5% with two different types of bipolar electrodes (TCRis, Versapoint) using saline, in hysteroscopic surgery. Fertil. Steril..

[6] Ting WH (2015). Safety and efficacy of manual syringe infusion of distending media for hysteroscopic procedures: a case-control study. Eur J Obstet Gynecol Reprod Biol..

[7] Wong AS (2014). Transcervical intralesional vasopressin injection compared with placebo in hysteroscopic myomectomy: a randomized controlled trial. Obstet. Gynecol..

[8] Chen SS, Lin AT, Chen KK, Chang LS (2006). Hemolysis in transurethral resection of the prostate using distilled water as the irrigant. J. Chin. Med. Assoc..

[9] Gupta K, Rastogi B, Jain M, Gupta PK, Sharma D (2010). Electrolyte changes: An indirect method to assess irrigation fluid absorption complications during transurethral resection of prostate: A prospective study. Saudi J. Anaesth..

[10] Khodorkovsky B, Cambria B, Lesser M, Hahn B (2014). Do hemolyzed potassium specimens need to be repeated?. J. Emerg. Med..

[11] Panovska Petrusheva A (2015). Evaluation of changes in serum concentration of sodium in a transurethral resection of the prostate. Pril. (Makedon Akad Nauk Umet Odd Med Nauki).

[12] Lee GY, Han JI, Heo HJ (2009). Severe hypocalcemia caused by absorption of sorbitol–mannitol solution during hysteroscopy. J. Korean Med. Sci..

[13] Agarwal R, Emmett M (1994). The post-transurethral resection of prostate syndrome: therapeutic proposals. Am. J. Kidney Dis..

[14] AAGL Advancing Minimally Invasive Gynecology Worldwide. et al. (2013). AAGL practice report: practice guidelines for the management of hysteroscopic distending media: (replaces hysteroscopic fluid monitoring guidelines. J Am Assoc Gynecol Laparosc. 2000;7:167–168). J. Minim. Invasive Gynecol..

[15] Indman PD (1998). Complications of fluid overload from resectoscopic surgery. J. Am. Assoc. Gynecol. Laparosc..

[16] Lin BL, Iwata Y, Liu KH (1994). Removing a large submucous fibroid hysteroscopically with the two-resectoscope method. J. Am. Assoc. Gynecol. Laparosc..

[17] Lin B, Akiba Y, Iwata Y (2000). One-step hysteroscopic removal of sinking submucous myoma in two infertile patients. Fertil. Steril..

[18] Rubino RJ, Lukes AS (2015). Twelve-month outcomes for patients undergoing hysteroscopic morcellation of uterine polyps and myomas in an office or ambulatory surgical center. J. Minim. Invasive Gynecol..

[19] Vitale SG (2017). Hysteroscopic morcellation of submucous myomas: a systematic review. Biomed. Res. Int..

[20] Emanuel MH (2015). Hysteroscopy and the treatment of uterine fibroids. Best Pract. Res. Clin. Obstet. Gynaecol..

[21] Arnold A, Ketheeswaran A, Bhatti M, Nesbitt-Hawes E, Abbott J (2016). A prospective analysis of hysteroscopic morcellation in the management of intrauterine pathologies. J. Minim. Invasive Gynecol..

[22] Haber K, Hawkins E, Levie M, Chudnoff S (2015). Hysteroscopic morcellation: review of the manufacturer and user facility device experience (MAUDE) database. J. Minim. Invasive Gynecol..

[23] Laganà AS (2017). Endometrial preparation with Dienogest before hysteroscopic surgery: a systematic review. Arch. Gynecol. Obstet..

[24] Kodama M (2013). Efficacy of dienogest in thinning the endometrium before hysteroscopic surgery. J. Minim. Invasive Gynecol..

[25] Tsai EM, Chiang PH, Hsu SC, Su JH, Lee JN (1998). Hysteroscopic resection of vaginal septum in an adolescent virgin with obstructed hemivagina. Hum. Reprod..

[26] Sethi N, Chaturvedi R, Kumar K (2012). Operative hysteroscopy intravascular absorption syndrome: A bolt from the blue. Indian J. Anaesth..

[27] Giacobbe V (2016). Otorrhagia and nosebleed as first signs of intravascular absorption syndrome during hysteroscopy: from bench to bedside. Kathmandu Univ. Med. J. (KUMJ)..

[28] Umranikar S (2016). BSGE/ESGE guideline on management of fluid distension media in operative hysteroscopy. Gynecol. Surg..

[29] Herbert A, Cruickshank JK, Laurent S, Boutouyrie P (2014). Reference Values for Arterial Measurements Collaboration. Establishing reference values for central blood pressure and its amplification in a general healthy population and according to cardiovascular risk factors. Eur. Heart J..

[30] Hasham F, Garry R, Kokri MS, Mooney P (1992). Fluid absorption during laser ablation of the endometrium in the treatment of menorrhagia. Br. J. Anaesth..

[31] Schulz, K. F., Altman, D. G., Moher, D. For the CONSORT Group. CONSORT 2010 statement: updated guidelines for reporting parallel group randomized trials. *Obstet. Gynecol*. **115**, 1063–1070 (2010). 10.1097/AOG.0b013e3181d9d421 20410783

