# Supplementary material for: Manual versus Pump Infusion of Distending Media for Hysteroscopic Procedures: A Randomized Controlled Trial
Source: Sci Rep. 2019 Oct 18;9:14943. doi: 10.1038/s41598-019-51252-3 (PMC6802079; doi:10.1038/s41598-019-51252-3)
Supplement: Supplementary file 2 — Research Protocol [file 41598_2019_51252_MOESM2_ESM.doc]

**Project Summary**

Various methods have been used in attempt to decrease the incidence of dilutional hyponatremia or the systemic absorption of the distending media, including automated fluid monitoring, intraoperative oxytocin or vasopressin use, or use of a bipolar resectoscope. This single-center randomized, controlled trial aimed at comparing the manual syringe infusion (MI) method with the conventional infusion pump (PI) method to clarify which infusion method will use less amount of distension medium.

The trial was performed at the Department of Obstetrics and Gynecology of Far Eastern Memorial Hospital (FEMH) from December 2013 and February 2017. All women who had agreed to participate the trial were randomly allocated to the MI or PI groups. Comparisons were made between the groups, including the volume of distending media used, perioperative data, changes of serum electrolytes and blood osmolarity.

It was anticipated that the MI group would be associated with lower amount of infused volume due to non-continuous fluid infusion, and thus had less impact on the changes of serum electrolytes and blood osmolarity.

**General Information**

**Protocol title:**

Manual versus Pump Infusion of Distending Media for Hysteroscopic Procedures: A Randomized Controlled Trial

**Clinical Trial Registration Number and date of registration:**

NCT02012010, 16/12/2013

**Number of IRB and date of approval:**

102124-F, 21/10/2013

**Name and address of the sponsor/funder:**

Far Eastern Memorial Hospital

No. 21, Sec. 2, Nanya S. Rd., Banqiao Dist., New Taipei City, Taiwan

**Name and title of the investigator:**

Principal investigator: Sheng-Mou Hsiao, MD

Co-investigator: Wan-Hua Ting, MD

**Address and telephone number of the research site:**

Department of Obstetrics and Gynecology, Far Eastern Memorial Hospital,

No. 21, Sec. 2, Nanya S. Rd., Banqiao Dist., New Taipei City, Taiwan

Tel.: 886-2-8966-7000, ext. 1424

Fax: 886-2-8966-5567

**Rationale & Background Information**

Hysteroscopy is frequently used for diagnosing abnormal uterine bleeding, endometrial polyp or submucous myoma. However, hysteroscopic procedure can cause morbidity and even mortality related to excessive absorption of distending media [1-4]. The electrolyte-free hypotonic distending media is often used to distend the uterine cavity for further diagnostic or therapeutic procedures. However, intravasation of hypotonic fluid may result in hyponatremia, cerebral edema, coma and even death [1-6]. Some measures were used to prevent the occurrence of hyponatremia events, such as automatic pump [7] or anesthesia method [8].

We have been using a manual syringe infusion method to infuse the distending media in hysteroscopic procedures for more than 2 years in our institute without significant morbidity, and this method is used by some physicians in America. This technique has some advantages, such as avoidance of continuous fluid infusion, more flexibility to allow temperately adequate distending pressure to explore the endometrial cavity, despite of the advantage of the need of an additional assistant to infuse the distending media using a syringe manually.

We are interested if this manual syringe infusion method can result in a less amount of distending media infusion than the conventional infusion pump method, and thus may reduce the occurrence of morbidity related to intravasation of large amount distending media [9]. Therefore, the aim of this study is to perform a randomized controlled trial of comparing the manual syringe infusion method with the conventional infusion pump method to clarify which infusion method will use less amount of distension medium.

#### Study Goals and Objectives

This study aims at comparing the amount of infused volume of the MI method with PI method in hysteroscopic procedures. The secondary objective of this study is to compare perioperative outcomes and postoperative changes in serum electrolytes and blood osmolarity between the MI and PI groups.

#### Study Design

This is an open-labelled, prospective, randomized controlled trial, conducted between December 2013 and February 2017. All the participants should provide written informed consent before receiving the interventions.

Inclusion criteria:

Women aged ≧20 years old with complaints of abnormal uterine bleeding, suspected endometrial lesion, endometrial polyps or submucosal myoma whom hysteroscopic exam or procedure is warranted for either diagnostic or therapeutic purpose

Exclusion criteria:

Woman aged <20 years old

Sample size:

To detect a difference of the amount of infused fluid, at least 25 subjects in each group were required to test the above hypothesis at a significance level of 0.05 and a power of 0.9.

**Methodology**

Randomization:

Women are allocated to the MI or PI groups in a randomized order based on computer-generated random numbers at a ratio of 1:1.

Before the surgery:

Baseline characteristics of all the participants are recorded (including age, parity, body mass index). For woman who is diagnosed with submucosal myoma, the largest diameter of the myoma is recorded based on sonographic picture. A blood sample is taken on the day of booking for analysis of complete blood count, serum electrolytes and blood osmolarity. Pre-operative pain scale (0–10) is determined by self-reporting using a visual analogue scale.

Surgical procedures:

The surgical procedures are performed by the corresponding author:

1) Under intravenous anesthesia, cervical dilation is done with Hegar uterine dilators, followed by insertion of a unipolar resectoscope with an outer diameter of 8 mm (Karl Storz, Tuttlingen, Germany) into the uterine cavity.

2) The uterine cavity is then infused with distilled water for inspection, resection, or ablation of tissue.

3) In the MI method, a 60 mL disposable syringe (BD Plastipak TM, BD Medical, County Louth, Ireland) is connected to the resectoscope via a 90 cm extension tube (Sigma, Sigma Medical Supplies Corp., Taiwan) and an assistant helps to pump the distilled water manually. Two 60 mL syringes are used at one time to minimize the waiting time required for refilling the syringe.

4) In the PI method, a continuous-flow fluid infusion pump device is used to deliver the fluid media at a constant in-flow pressure of 70-100 mmHg, depending on the visibility of the surgical view.

5) A large collecting bag is tucked beneath the woman’s gluteal region and secured to the surgeon’s gown to capture fluid spilled from the cervix and the resectoscope.

6) The total volumes of infused fluid and outflow fluid are recorded, as well as the operating time and estimated blood loss.

After the surgery:

A second blood sample is taken after the surgery for analysis of complete blood count, serum electrolytes and blood osmolarity. The immediate post-operative pain scale (0–10) is determined by self-reporting using a visual analogue scale when the woman recovers from anesthesia in the recovery room.

Measurements:

The fluid deficit is calculated by subtracting the total volume of collected fluid media from the total infused volume. Changes of serum electrolytes, blood osmolarity, hemoglobin and pain scores are determined by subtracting the preoperative measurements from the postoperative ones.

#### Safety Considerations

Hysteroscopic procedures is associated with potential surgical risks regardless the type of infusion method used, including uterine rupture, uncontrolled bleeding, water intoxication, hyponatremia, brain edema or even death. This trial advocates the general principle of hysteroscopic procedure that emphasizes strict monitoring of operation time and the amount of fluid deficit in order to minimize the event of fluid overload. In case of emergent situation such as water intoxication or hyponatremia, the woman will be admitted to the gynecological ward and put under close surveillance until her clinical condition stabilizes.

The two infusion methods used in this trial have been practiced for years in this institution, and thus will not confer additional surgical risks to the general hysteroscopic procedures. All women will be given details of the surgical procedures, and they will be requested to return to clinic after the surgery for subsequent follow ups.

#### Follow-Up

All women are instructed to return to the clinic one week after the intervention for pathological report, as well as evaluation of their health status. Subsequent outpatient follow-up will be booked in advance if deemed necessary, i.e persistent bleeding or pain.

#### Data Management and Statistical Analysis

All the data collected will be kept confidential and anonymous, and the results reported in the aggregate. Information on individuals is only used by the principal and co-investigators for the purpose of the study, it will not be shared with the third party, except when applicable by law. Each individual will be designated a research number as the substitute for her name.

The research results will be analyzed and published in scientific journal. Both the Wilcoxon rank-sum test and the Chi-square test will be employed for statistical analyses using STATA software (Version 11.0; Stata Corp, College Station, TX, USA). A p-value of less than 0.05 is considered statistically significant.

A previous study reported an infused volume of 1117±712 mL in the MI group and 2216±1502 mL in the PI group [10]. To detect a difference of the amount of infused fluid, we conducted a test with a significance level of 0.05 and a power of 0.9, and concluded that at least 25 subjects in each group were required to test the hypothesis.

#### Expected Outcomes of the Study

It is anticipated by comparing the differences in pre-, peri- and post-operative parameters of study, a conclusion can be drawn regarding the efficacy of the MI method in reducing hysteroscopic procedures related complications, as well as to investigate the impact of fluid absorption on serum electrolytes and blood osmolarity.

#### Dissemination of Results and Publication Policy

The results of the study will be written into a manuscript and submitted for publication in English international journal by both the principal and co-investigators.

#### Duration of the Project

Recruitment of participants started since October 2013, it is estimated that at least 12~18 months is required to collect adequate number of cases.

| **Date** | **Event** |
| --- | --- |
| 2013/12/06 | Recruitment of 1^st^ case |
| 2013/11/05~2014/04/11 | Ongoing recruitment (number of cases: 20) |
| 2014/04/12~2014/10/03 | Ongoing recruitment (number of cases: 14) |
| 2014/10/04~2015/04/16 | Ongoing recruitment (number of cases: 17)  Extension of project in order to recruit more participants (target: 100 cases) to improve the validity of research as well as to minimize research bias |
| 2015/04/17~2015/12/26 | Ongoing recruitment (number of cases: 23) |
| 2015/12/27~2016/12/31 | Ongoing recruitment (number of cases: 23) |
| 2017/01/01~2017/08/25 | Termination of project (total number of cases: 101) |

#### Project Management

Dr Sheng-Mou Hsiao is the principal investigator of the project, responsible for the recruitment of participants, explanation of the informed consent forms, as well as the main surgeon of all the hysteroscopic procedures.

Dr Wan-Hua Ting is the co-investigator, who is responsible to collect all the baseline data of the participants and all the necessary measurements required for the analysis, as well as to serve as the assistant of the hysteroscopic procedures performed by the principal investigators.

#### Ethics

This study has been approved by the Research Ethics Committee of Far Eastern Memorial Hospital. The principal investigator will explain the research protocol to eligible women during office visit, and invite them to join the research. Eligible women will be provided with a copy of informed consent form, and given adequate time to discuss with their family members. Once they have fully understood all the content of the informed consent form, they will be asked to sign the form and hand it back to the principal investigator.

Participants may stop participating in the research at any time they choose. All of their rights will still be respected and their treatment at the institution will not be affected in any way.

#### Informed Consent Forms

Please refer to the supplementary files for the informed consent forms in both the Chinese and English language (Suppl. File No. 1, Suppl. File No. 2).

**Budget**

This project is supported by the Far Eastern Memorial Hospital, with the total amount of funding NT220,000. Expenses reimbursement is detailed as below:

| **Date** | **Subject** | **Amount (NTD)** |
| --- | --- | --- |
| 2014/03/18 | Salary and wages for project assistant (March) | 43,625 |
| 2014/04/14 | Salary and wages for project assistant (April) | 44,555 |
| 2014/05/02 | Microscope slides | 1,050 |
| 2014/08/29 | Fees for research papers | 50 |
| 2014/10/01 | Stationery | 611 |
| 2014/12/29 | Stationery | 504 |
| 2015/05/11 | Access fee to National Health Insurance Database | 129,000 |
| 2015/05/12 | Photocopy | 254 |
| 2015/05/15 | Stationery | 320 |
|  | **Total Amount** | 219,969 |

**Other support for the Project:** Not applicable

**Collaboration with other scientists or research institutions:** Not applicable

**Links to other projects:** Not applicable

**Curriculum Vitae of investigators:** Please refer to the supplementary files for the curriculum vitae of the investigators (Suppl. File No. 3, Suppl. File No. 4)

**Financing and Insurance:** Not applicable

**References**

1. Yang BJ, Feng LM. [Symptomatic hyponatremia and hyperglycemia complicating hysteroscopic resection of intrauterine adhesion: a case report.](http://www.ncbi.nlm.nih.gov/pubmed/22613661) Chin Med J (Engl) 2012;125:1508-10.
2. Woo YC, Kang H, Cha SM, Jung YH, Kim JY, Koo GH, Park SG, Baek CW. [Severe intraoperative hyponatremia associated with the absorption of irrigation fluid during hysteroscopic myomectomy: a case report.](http://www.ncbi.nlm.nih.gov/pubmed/22137519) J Clin Anesth 2011;23:649-52.
3. Jo YY, Jeon HJ, Choi E, Choi YS. [Extreme hyponatremia with moderate metabolic acidosis during hysteroscopic myomectomy -A case report-.](http://www.ncbi.nlm.nih.gov/pubmed/21738849) Korean J Anesthesiol 2011;60:440-3.
4. Wegmüller B, Hug K, Meier Buenzli C, Yuen B, Maggiorini M, Rudiger A. [Life-Threatening Laryngeal Edema and Hyponatremia during Hysteroscopy.](http://www.ncbi.nlm.nih.gov/pubmed/21541246) Crit Care Res Pract 2011;2011:140381.
5. [Sethi N](http://www.ncbi.nlm.nih.gov/pubmed?term=Sethi N%5BAuthor%5D&cauthor=true&cauthor_uid=22701213), [Chaturvedi R](http://www.ncbi.nlm.nih.gov/pubmed?term=Chaturvedi R%5BAuthor%5D&cauthor=true&cauthor_uid=22701213), [Kumar K](http://www.ncbi.nlm.nih.gov/pubmed?term=Kumar K%5BAuthor%5D&cauthor=true&cauthor_uid=22701213). Operative hysteroscopy intravascular absorption syndrome: A bolt from the blue. [Indian J Anaesth](http://www.ncbi.nlm.nih.gov/pubmed/22701213) 2012;56:179-82.
6. [Murakami T](http://www.ncbi.nlm.nih.gov/pubmed?term=Murakami T%5BAuthor%5D&cauthor=true&cauthor_uid=15916657), [Tamura M](http://www.ncbi.nlm.nih.gov/pubmed?term=Tamura M%5BAuthor%5D&cauthor=true&cauthor_uid=15916657), [Ozawa Y](http://www.ncbi.nlm.nih.gov/pubmed?term=Ozawa Y%5BAuthor%5D&cauthor=true&cauthor_uid=15916657), [Suzuki H](http://www.ncbi.nlm.nih.gov/pubmed?term=Suzuki H%5BAuthor%5D&cauthor=true&cauthor_uid=15916657), [Terada Y](http://www.ncbi.nlm.nih.gov/pubmed?term=Terada Y%5BAuthor%5D&cauthor=true&cauthor_uid=15916657), [Okamura K](http://www.ncbi.nlm.nih.gov/pubmed?term=Okamura K%5BAuthor%5D&cauthor=true&cauthor_uid=15916657). Safe techniques in surgery for hysteroscopic myomectomy. [J Obstet Gynaecol Res](http://www.ncbi.nlm.nih.gov/pubmed/15916657) 2005;31:216-23.
7. Kumar A, Kumar A. [New hysteroscopy pump to monitor real-time rate of fluid intravasation.](http://www.ncbi.nlm.nih.gov/pubmed/22425143) J Minim Invasive Gynecol 2012;19:369-75.
8. Bergeron ME, Ouellet P, Bujold E, Cote M, Rhéaume C, Lapointe D, Beaudet C, Lemyre M, Laberge P. [The impact of anesthesia on glycine absorption in operative hysteroscopy: a randomized controlled trial.](http://www.ncbi.nlm.nih.gov/pubmed/21788316) Anesth Analg 2011;113:723-8.
9. AAGL Advancing Minimally Invasive Gynecology Worldwide, Munro MG, Storz K, Abbott JA, Falcone T, Jacobs VR, Muzii L, Tulandi T, Indman P, Istre O, Jacobs VR, Loffer FD, Nezhat CH, Tulandi T. [AAGL Practice Report: Practice Guidelines for the Management ofHysteroscopic Distending Media: (Replaces Hysteroscopic Fluid Monitoring Guidelines. J Am Assoc Gynecol Laparosc. 2000;7:167-168).](http://www.ncbi.nlm.nih.gov/pubmed/23465255)
10. Ting, W.H. et al. Safety and efficacy of manual syringe infusion of distending media for hysteroscopic procedures: a case-control study. Eur J Obstet Gynecol Reprod Biol. 191, 112–115 (2015).

***Suppl. File No. 1***

**亞東紀念醫院**

**受試者說明及同意書**

（為保護病人權益，請將容易造成受試者危險或警示字句標示出來）

（本書表應由計畫主持人親自向受試者說明詳細內容，並請受試者經過慎重考慮後方得簽名）

您被邀請參與此研究。本表格提供您有關本研究之相關資訊，研究主持人或其他協同主持醫師將會為您說明研究內容並回答您的任何疑問。

| 計畫名稱：比較手動空針給水及傳統幫浦給水方式以執行子宮鏡手術:一隨機分派試驗。 | | | | | | | |
| --- | --- | --- | --- | --- | --- | --- | --- |
| 主要主持人： | 蕭聖謀醫師 | 聯絡電話： | | | 0919-302632 | | |
| 協同主持人： | 陳奐樺醫師 |  | | |  | | |
|  |  |  | |  | | | |
|  |  | | | | | | |
| 受試者姓名： |  | 性別： |  | | | 年齡： |  |
|  |  |  |  | | |  |  |
| 聯絡通訊地址： |  | | | | | | |
|  |  | | | | | | |
| 電話： |  | 病歷號碼： | | |  | | |
|  |  |  | | |  | | |
| **一、研究目的：**比較手動空針給水及傳統幫浦給水方式以執行子宮鏡手術，探討人工給水的方式是否較傳統幫浦給水方式可減少給水量，進而推論出可減少手術過程中所引發的相關併發症。  **二、研究方法與程序：**   1. 收案條件：大於二十歲之成年女性病患有異常子宮出血、子宮內膜息肉或肌瘤等，而須做子宮鏡檢查或手術者。 2. 排除條件：小於二十歲之女性病患。   隨機分派方式採用電腦給序方式，決定病人在何組。在控制組則是接受傳統之幫浦給水方式；而實驗組則是接受利用手動50ml空針給水方式。其餘子宮鏡之步驟就如同常規之手術方式。術後兩組皆加抽血液鈉及血紅素之數值。手術給水量及收集袋中之水量皆須記錄。  傳統之幫浦給水方式乃是利用幫浦定壓方式送水至子宮鏡，而進入子宮腔。優點是水壓固定、持續給水。缺點是傳到子宮腔之水壓可能不足，有時不易看清楚子宮腔；須調高水壓。  利用手動50ml空針給水方式需一助手，利用50 mL之針筒把水打入子宮鏡中，而進入子宮腔。優點是可以有較足夠之瞬間水壓看清楚子宮腔，因此子宮腔之水壓並非恆定，避免子宮腔一直處在高水壓之環境下；同時因非持續給水，總給水量可能較少。缺點是可能子宮腔瞬間水壓較大，術後可能下腹部較不適。  不管何種給水方式來執行子宮鏡手術都有潛在之副作用與風險。例如:子宮穿孔、水中毒、低血鈉、甚至腦水腫昏迷等。本研究依循一般子宮鏡執行之原則嚴格計算手術時間及子宮腔進出水量，以求減少水中毒之風險。若產生緊急狀況(例如水中毒)，則必須住院予以支持性療法，以度過危險期。  預計收案人數為每組各五十人。  **三、身心上可能導致之副作用、不適或危險：**本研究使用二種給水方式，並非新的方式，已行之有年，一般不會對一般子宮鏡手術方式有額外增加手術風險。  **四、預期研究效果：**得知哪一種給水方式在執行子宮鏡手術有較少的給水量，進而推論出可減少手術術後對病人所引發的相關併發症。  **五、其他可能之治療方法選擇及說明**：依受試者的病情狀況，使用常規性的治療方法。若您不同意參與本研究，也有自行以選擇不同給水方式施行子宮鏡手術之權利。  **六、其他可能之損失或利益：**   - 可能利益：   - 1. 研究結果將能使醫師替您及其他需接受子宮鏡患者提供更進一步的諮詢、治療。     2. 本研究結果可能提供新的資料，而使您或其他接受子宮鏡之患者受益。 - 可能損失：   但由於本研究使用二種給水方式，並非新的方式，已行之有年，一般不會對一般子宮鏡手術方式有額外增加手術風險。  **子宮鏡手術併發症少見。相對常見子宮鏡手術併發症包括: 子宮流血過多、子宮穿孔、子宮頸裂傷、骨盆腔發炎、水中毒等。若水中毒可能導致低血鈉、腦水腫昏迷等。**  **七、參加本研究計畫受試者個人權益將受到保護：**  如依本研究所訂臨床試驗計畫，因發生不良反應造成損害，由亞東紀念醫院負補償責任。但本受試者同意書上所記載之可預期不良反應，不予補償。  如依本研究所訂試驗計畫而引發之身體、心理上之不良反應、副作用或傷害，本醫院及主持人將提供受試者專業醫療照顧及醫療諮詢。您不必負擔治療不良反應或損害之必要醫療費用。  除法定賠償及醫療照顧外，本研究不提供其他形式之賠償或補償。若您不願意接受這樣的風險，請勿參加試驗。  您不會因為簽署本同意書，而喪失在法律上的任何權利。  （本研究未投保責任保險。）  **八、受試者具有參與研究及於研究中途撤回同意之自由選擇權：**  如您選擇參加，亦可隨時撤回同意，中止研究之參與，而此項決定並不影響醫師對您之醫療照護。如您因任何理由欲中止研究之參與，請聯絡研究主持人 蕭聖謀醫師 ，24小時緊急聯絡電話： 0919302632  **九、受試者權利：**  試驗過程中，與你(妳)的健康或是疾病有關，可能影響你(妳)繼續接受臨床試驗意願的任何重大發現，都將即時提供給你(妳)。  如果你(妳)在試驗過程中對試驗工作性質產生疑問，對身為患者之權利有意見或懷疑因參與研究而受害時，可與本院之人體試驗審議委員會聯絡請求諮詢，其電話號碼為：89667000 分機2152。  **十、機密性：**  亞東紀念醫院將在法律所規範之程度內將您的資料視為機密，您亦瞭解衛生署與本院人體試驗審議委員會皆有權檢視您的資料並會遵守保密之倫理。  所有的資料將會依法作為機密處理。對於檢查的結果及醫師診斷，計畫主持人將持保密的態度，且將受試者進行編碼，以一個研究號碼取代姓名，且電腦加密。保存期限為研究結束後10年。除了有關機構依法調查外，計畫主持人會小心維護隱私。本研究之結論將力求於國際英文期刊上發表。  **十一、利益衝突：**本試驗無廠商參予計畫之研擬  **十二、剩餘檢體使用部分：**不適用本研究  **十三、簽章**  (一) 主要主持人、協同主持人、代理主持人或研究護士已詳細解釋並回答有關本研究計畫中上述研究方法的性質與目的，及可能產生的危險與利益。  □主要主持人/□協同主持人/□研究護士/□訪查員  簽章：_______________ 日期：_______________  (二) 受試者或法定代理人已詳細瞭解上述研究方法及其所可能產生的危險與利益，有關本試驗計畫的疑問，業經計畫主持人詳細予以解釋。本人同意接受為臨床試驗計畫的自願受試者。  **受試者** 簽章：_______________ 日期：_______________  **法定代理人** 簽章：_______________ 日期：_______________  ＊**受試者為7至20歲青少年已具部分行為能力者，受試者與法定代理人應同時簽名。**  ＊**法定代理人應同時出示相關證明文件以供試驗主持人確認身份。**  (三) 有同意權人  姓名：_______________ 關係：_______________  身份證字號： **______________** 聯絡電話**：________________**  日期：**______________**  **＊受試者雖非無行為能力或限制行為能力者，但因意識混亂或有精神與智能障礙，而無法進行有效溝通和判斷時，由有同意權之人為之。前項有同意權人為配偶及直系親屬或同居親屬，簽署時應同時出示相關證明文件以供試驗主持人確認身份。**  (四) 見證人  姓名：_______________ 身份證字號：**______________**  聯絡電話**：_______________** 日期：_______________  **＊受試者、法定代理人或有同意權之人皆無法閱讀時，應由見證人在場參與所有有關受試者同意書之討論。試驗相關人員不得為見證人。** | | | | | | | |

***Suppl. File No. 2***

**Far Eastern Memorial Hospital**

**Informed Consent Form (English Version)**

You have been invited to join the research. This form provides you with information regarding the research, the investigators will explain the content of the research and answer all your questions.

| **Research Title：**Manual versus Pump Infusion of Distending Media for Hysteroscopic Procedures: A Randomized Controlled Trial | | | | | | | |
| --- | --- | --- | --- | --- | --- | --- | --- |
| **Principal investigator：** | Dr Sheng-Mou Hsiao | | | | **Tel:** | | 0919-302632 |
| **Co-investigator：** | Dr Wan-Hua Ting | | | |  | |  |
|  |  |  |  | | | | |
|  |  | | | | | | |
| **Name of the participant:** |  | **Sex:** | |  | | **Age:** |  |
|  |  |  | |  | |  |  |
| **Address:** |  | | | | | | |
|  |  | | | | | | |
| **Tel:** |  | **Chart No.:** | | | | |  |
|  |  |  | | | | |  |
| **Purpose:**  To validate if manual infusion (MI) of distending media during hysteroscopic procedure is associated with less infused volume and thus reduce the incidence of surgical related complications, when compared with pump infusion (PI) method  **Methodology:**   - Inclusion criteria: Woman aged ≧20 years old with complaints of abnormal uterine bleeding, suspected endometrial lesion, endometrial polyps or submucosal myoma whom hysteroscopic exam or procedure is warranted for either diagnostic or therapeutic purpose - Exclusion criteria: Woman aged <20 years old   Allocation of the group is done based on computer-generated random numbers. The control group will be using conventional PI method; whereas the experimental group will be using MI method with a 60mL disposable syringe. The rest of the surgical procedures are performed as usual. Blood sample will be drawn before and after the surgery for analysis of serum electrolytes, blood osmolarity and hemoglobin level. The total infused and collected fluid will be recorded.  The conventional PI method utilizes a simple pump device that dispend distending media into the uterine cavity via the resectoscope at a constant pressure. The advantage of the PI method is its continuous and stable flow of fluid, but it might be associated with inadequate intrauterine pressure, obscuring the surgical view, and thus requires the adjustment of the pressure.  In the MI method, an assistant will help to infuse the distending media using a 60mL disposable syringe. The advantage of the MI method is the transient high intrauterine pressure that enables the surgeon to identify the lesion, thus the uterine cavity is not under constant high intrauterine pressure all the time. Due its noncontinuous fluid infusion nature, the total infused volume is probably relatively small. However, participant might have lower abdominal discomfort due to the transient high intrauterine pressure.  Hysteroscopic procedures is associated with potential surgical risks regardless the type of infusion method used, including uterine rupture, uncontrolled bleeding, water intoxication, hyponatremia, brain edema or even death. This research advocates the general principle of hysteroscopic procedure that emphasizes strict monitoring of operation time and the amount of fluid deficit in order to minimize the event of fluid overload. In case of emergent situation such as water intoxication or hyponatremia, the participant will be admitted to the gynecological ward and put under close surveillance until her clinical condition stabilized.  Estimated number of recruits is at least 50 in each group.    **Side Effects:**  This research utilizes two methods for infusing distending media, these methods have been practiced for years in this institution, and thus will not confer additional surgical risks to the general hysteroscopic procedures.  **Expected Outcome:**  To determine which method is associated with less amount of infused volume, and this possibly means a reduction in surgical related complications  **Alternatives to Participating:**  If you do not agree to take part in the research, you may choose whichever infusion method you prefer.  **Benefits and Risks:**  Benefits:   - The result of the research enables clinicians to provide evidence-based recommendations on hysteroscopic procedures during counseling. - The new findings of this research may provide new information that is beneficial for women who will be receiving hysteroscopic procedures in the future.   Risks:  Complications related to hysteroscopic procedures are rare. However, you should still be aware of the possibility of excessive intra-operative blood loss, uterine rupture, cervical laceration, infection, water intoxication. Water intoxication might lead to hyponatremia, brain edema and coma. We will try to decrease the chances of these events occurring, but if something unexpected happens, we will provide you with all the necessary medical attention and treatment.  **Protection of Personal Rights**   - In case of any adverse events, Far Eastern Memorial Hospital will take the full responsibility, with exceptions to the expected adverse event as stated in this form. - If you have any physical or psychological adverse effects related to this research protocol, you will be provided with professional medical care and consultation without additional cost. - With exception to the statutory compensation and medical care, we do not provide any other form of compensation. If you do not wish to take the risk, please do not take part. - You will not lose any rights in the law if you sign this informed consent form. - This research has no liability insurance.   **Right to Refuse or Withdraw**  You may stop participating in the research at any time you choose. It is your choice and all of your rights will still be respected. Your treatment at this institution will not be affected in any way. If you wish to withdraw, please contact the principal investigator Dr Sheng-Mou Hsiao (Tel: 0919302632)  **Subject’s Rights**   - Throughout the research, you will be provided with any information or findings that are relevant to your health or disease status. You may decide whether to continue or withdraw from the research. - If you are doubtful about the nature of the research, you may exert your rights by contacting the member of the institutional review board for further inquiries, Tel.no 89667000#2152.   **Confidentiality**   - The information that we collect from this research project will be kept confidential. However, you should acknowledge the fact that the Institutional Review Board of this institution, as well as the Ministry of Health and Welfare have access to the information but they will abide by the ethics of confidentiality. - Information about you that will be collected during the research will be put away and no-one but the researchers will be able to see it. Any information about you will have a number on it instead of your name. Only the researchers will know what your number is and we will lock that information up with computer encryption. All the relevant data will be stored for another 10 years after the completion of the research. We will analyze the data and publish the result in international English journal.   **Conflict of Interest**  There is no sponsorship from any manufacturers except the institution.  **Usage of the Remaining Pathological Specimen**  Not applicable to this research  **Signature**   - **Statement by the Researcher Taking Consent**   I have accurately read out the information sheet to the potential participant, and to the best of my ability made sure that the participant understands the research protocol. I confirm that the participant was given an opportunity to ask questions about the study, and all the questions asked by the participant have been answered correctly and to the best of my ability.  **Signature of Researcher /person taking the consent**__________________________  **Date** ___________________________   - **Statement by the Participant**   I have read the foregoing information, or it has been read to me. I have had the opportunity to ask questions about it and any questions that I have asked have been answered to my satisfaction. I consent voluntarily to participate as a participant in this research.  **Signature of Participant** ___________________­­­___ **Date** ___________________________  **Signature of Legal Guardian** ___________________ **Date** ___________________________   - **Witness**   If illiterate, a literate witness must present to take part in all the discussion related to this informed consent form and sign. This person should have no connection to the research team.  **Signature of Witness** ___________________­­­_____ **ID number**___________________________  **Contact No.** ___________________ **Date** ___________________________ | | | | | | | |
|  | | | | | | | |

***Suppl. File No. 3***

**Curriculum Vitae of Principal Investigator**

**Personal Particulars**

Last Name : Hsiao Given Name : Sheng-Mou

Email : smhsiao2@gmail.com

Date of Birth : 04 March 1969

Nationality : Taiwan

Address : No.21, Nanya South Road Section 2, Banqiao District, New Taipei City, Taiwan

Contact No. : (Office) +886-2-7728-1818 (Mobile Taiwan) +886-919-302632

Fax No. : (Office) +886-2-7728-2446

**Academic Qualification**

1. Title : Bachelor of Medicine

Course duration : Sept 1989~Jun 1996

Country of Study : Taiwan

Institution Attended : National Taiwan University College of Medicine

1. Title : Master of Clinical Medicine

Course duration : Sept 2000~Jun 2002

Country of Study : Taiwan

Institution Attended : National Taiwan University College of Medicine, Graduate Institute of

Clinical Medicine

**Specialty**

1. Gynae-oncology **(2)** Gynae-urology

**Career**

1. Posting : Chief

Department : Department of Obstetrics and Gynecology, Far Eastern Memorial Hospital

Duration : August 2009~till now

1. Posting : Associate professor (Part-time)

Department : National Taiwan University College of Medicine

Duration : August 2016~till now

1. Posting : Jointly appointed associate professor

Department : Graduate School of Biotechnology and Bioengineering, Yuan Ze University

Duration : August 2016~till now

***Suppl. File No. 4***

**Curriculum Vitae of Co-investigator**

**Personal Particulars**

Last Name : Ting Given Name : Wan-Hua (Stella)

Email : stellatingwh@yahoo.com / stellatingwh@gmail.com

Date of Birth : 20 November 1985

Nationality : Malaysian

Address : No.21, Nanya South Road Section 2, Banqiao District, New Taipei City, Taiwan

Contact No. : (Office) +886-2-7728-2426 (Mobile Taiwan) +886-955-855120

Fax No. : (Office) +886-2-7728-2446

**Academic Qualification**

1. Title of Degree : Bachelor of Medicine

Course duration : Sept 2004~Jun 2011

Country of Study : Taiwan

Institution Attended : Kaohsiung Medical University

**Specialty**

**(1)** Gynae-oncology **(2)** Gynae-urology

**Career**

1. Posting : Fellowship training in Gynae-oncology and Gynae-urology

Department : Department of Obstetrics and Gynecology, Far Eastern Memorial Hospital

Duration : August 2016~till now

1. Posting : Residency training in Obstetrics and Gynecology

Department : Department of Obstetrics and Gynecology, Far Eastern Memorial Hospital

Duration : August 2012~July 2016
